# Biophysical basis for convergent evolution of two veil-forming microbes

**DOI:** 10.1098/rsos.150437

**Published:** 2015-11-11

**Authors:** Alexander P. Petroff, Alexis L. Pasulka, Nadine Soplop, Xiao-Lun Wu, Albert Libchaber

**Affiliations:** 1 Laboratory of Experimental Condensed Matter Physics, The Rockefeller University, New York City, NY 10065, USA; 2 Electron Microscopy Resource Center, The Rockefeller University, New York City, NY 10065, USA; 3 Division of Geological and Planetary Sciences, California Institute of Technology, Pasadena, CA 91125, USA; 4 Department of Physics and Astronomy, University of Pittsburgh, Pittsburgh, PA 15260, USA

**Keywords:** convergent evolution, hydrodynamics, collective dynamics

## Abstract

Microbes living in stagnant water typically rely on chemical diffusion to draw nutrients from their environment. The sulfur-oxidizing bacterium *Thiovulum majus* and the ciliate *Uronemella* have independently evolved the ability to form a ‘veil’, a centimetre-scale mucous sheet on which cells organize to produce a macroscopic flow. This flow pulls nutrients through the community an order of magnitude faster than diffusion. To understand how natural selection led these microbes to evolve this collective behaviour, we connect the physical limitations acting on individual cells to the cell traits. We show how diffusion limitation and viscous dissipation have led individual *T. majus* and *Uronemella* cells to display two similar characteristics. Both of these cells exert a force of approximately 40 pN on the water and attach to boundaries by means of a mucous stalk. We show how the diffusion coefficient of oxygen in water and the viscosity of water define the force the cells must exert. We then show how the hydrodynamics of filter-feeding orient a microbe normal to the surface to which it attaches. Finally, we combine these results with new observations of veil formation and a review of veil dynamics to compare the collective dynamics of these microbes. We conclude that this convergent evolution is a reflection of similar physical limitations imposed by diffusion and viscosity acting on individual cells.

## 1. Introduction

Microbes living in the bottom millimetre of marshes typically rely on chemical diffusion to transport nutrients to the cell [1,2]. Because diffusion over this distance is slow compared to the metabolic rates of microbes, many cells become diffusion limited [3–7]. Microbes have evolved a remarkable array of traits that allow cells to mitigate this physical limitation [5,7–10].

**Figure 1.**
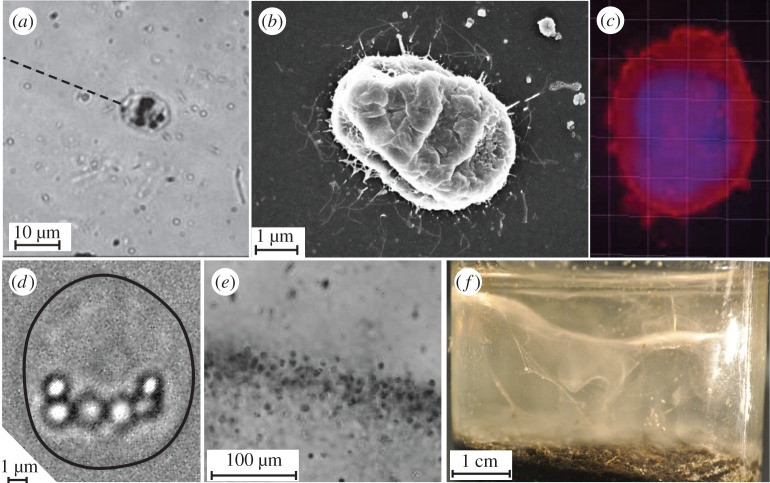
*Thiovulum majus* is a sulfur-oxidizing bacteria. (*a*) Individual cells attach to surfaces by exuding a mucous stalk. The stalk is invisible at magnification. Its position is shown with a dashed line. (*b*) Many of the flagella (thin white lines) surrounding a *T. majus* can be imaged with a scanning electron microscope. Note that this cell shrank during the ethanol dehydration. (*c*) Staining *T. majus* with DAPI (violet) and Dil (red) allows one to visualize the distribution of DNA and the cell membrane, respectively. (*d*) Sulfur globules within the cell posterior appear as bright micrometre-scale inclusions. The cell outline is shown in black. (*e*) A band of *T. majus* cells observed under a microscope. Note that the cells are organized into a dense front of cells. (*f*) The veil appears in an enrichment culture as a contorted white membrane extending the diameter of the serum bottle.

**Figure 2.**
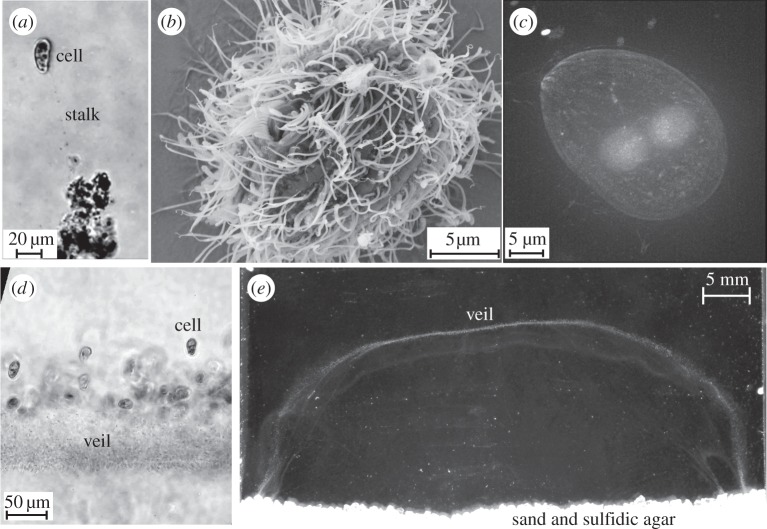
*Uronemella* is a genus of ciliates. (*a*) Individual cells exude a thin mucous stalk. Several small particles can be seen stuck to this stalk. (*b*) An electron micrograph shows that individual cells are covered in several hundred cilia. The cell uses these cilia to pull water past the cell. Like the *T. majus* cell shown in figure 1*b*, the cell size has shrunk considerably as a result of desiccating the cells. (*c*) DAPI stained *Uronemella* overlain on dark field. Two spherical inclusions show the nuclei. (*d*) A front of *Uronemella* cells are shown with a veil. As the stalks of neighbouring cells become entwined, they form a veil. Note that nearly all of the cells are attached on the same side of the front. (*e*) An *Uronemella* veil grown between two microscope slides separated by 1 mm. The veil appears as a white U-shaped line. One millimolar sulfide diffusing from agar at the base of the chamber mixes with oxygen in the media, providing the sole energy source for the enrichment culture.

**Figure 3.**
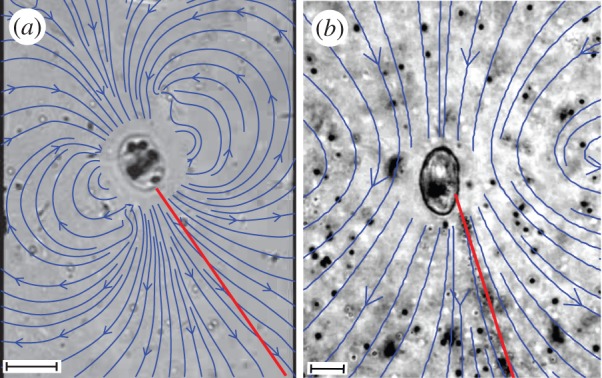
Both *T. majus* and *Uronemella* anchor to a surface with a mucous stalk (red line) and they exert a force on the fluid to generate a flow. Blue arrows show the stream lines of water as it is pulled past the cell. These stream lines were reconstructed from the motion of tracer particles around the cell, as previously described [19]. The vortex to either side of the cell is generated by the back flow from the nearby cover slip [20,21]. (*a*) *Thiovulum majus* generates a flow by rotating flagella. (*b*) *Uronemella* generates the flow by synchronously beating its cilia. Scale bars, (*a*,*b*) 10 μm.

One of the most dramatic strategies to draw nutrients from the environment is displayed by both the bacterium *Thiovulum majus* [11–13] (figure 1) and the ciliate *Uronemella spp.* [14,15] (figure 2). These microbes have separately evolved the ability to form communities that generate large-scale fluid flows which transport oxygen to cells forty times faster than diffusion [9,16]. To do so, cells first accumulate into a band at a particular oxygen concentration [17,18]. Within the band, a cell produces a 10–100 μm-long mucous stalk, which it uses to attach to a surface or the stalks of neighbouring cells [15,17]. Once anchored in place, the cell exerts a force that pulls water past the cell, as shown in figure 3. As many cells attach to one another (figures 1*c* and 2*c*), their stalks become entwined to form an elastic sheet called a ‘veil’ that can be several centimetres in diameter (figures 1*d* and 2*d*) [9,12,16,22–24]. Cells attach to one side of the veil and align their bodies to pull oxygen-rich water through the veil [16,17]. Neighbouring cells are separated from one another by only a few body lengths, corresponding to a surface density of approximately 10^6^ *T. majus* cm^−2^ or approximately 10^5^ *Uronemella* cm^−2^.

The veil provides two benefits to the constituent cells. First, it provides a permeable surface to which cells attach. When cells anchor to an impermeable surface, such as a sand grain, the flow created by a cell is slowed as it passes over its surroundings. By forming a permeable surface separated from the marsh bottom, cells can avoid the screening effect of surfaces and thus produce a greater flow of water. Second, the veil provides a substrate on which cells organize to produce a macroscopic flow. When cells are uniformly distributed on a sheet, they only stir their environment on the scale of a cell. Attachment and detachment of cells from the veil generate millimetre-scale density fluctuations [16,23,24] that produce a large-scale convective flow [9,16,22].

The remarkably similar behaviour [18] displayed by these microbes is surprising given that *T. majus* and *Uronemella spp.* are phylogenetically distinct. Table 1 provides a list of the characteristics of these two microbes. *Thiovulum majus* (figure 1), a 10 μm sulfur-oxidizing bacterium [25,26], is the second-fastest bacterium known [27–29]. This species of bacteria lives at the bottom of salt marshes, oxidizing sulfide—produced as a by-product of anaerobic decomposition in the underlying sediment—with oxygen from the overlying water [9,12]. *Thiovulum majus* attaches to a surface by means of a mucous stalk and exerts a force by rotating several hundred flagella, which cover the cell surface [11]. It is not understood how *T. majus* is able to use the typical flagellar proteins to swim so much faster than other bacteria [30]. By contrast, members of the genus *Uronemella* (figure 2) are found in mats of sulfur-oxidizing bacteria, which they consume [18]. These 25 μm ciliates exude a mucous stalk, which attaches to the cell along an elongated cilium [15,18]. Once anchored to a surface, the cell exerts a force by beating its cilia, several hundred of which cover the cell in lines extending between the anterior and posterior poles of the cell [15].

**Table 1.** Characteristics of the bacterium *T. majus* and ciliate *Uronemella*.

	*T. majus*	*Uronemella*
size	5–20 μm	25 μm
swimming speed	400–600 μm s^−1^	250 μm s^−1^
force	40 pN	50 pN
force generation	∼100 flagella	∼100 cilia
stalk growth	0.65 μm s^−1^	1.5 μm s^−1^
cell density on veil	10^6^ cells cm^−2^	10^5^ cells cm^−2^
optimal oxygen concentration	4% atm	7% atm

Because natural selection acts on individual genotypes, it is unclear how microbes evolve the ability to benefit from collective behaviour [31–35]. Should one conclude that *T. majus* and *Uronemella* have both evolved a suite of adaptations that allow cells to coordinate their actions even in the presence of many of other microbial species? Here, we combine past observations with new experiments and analysis to highlight how the similar collective dynamics displayed by these veil-forming microbes arise from the behaviours of individual cells to mitigate diffusion limitation. The formation of a veil and the generation of a large-scale flow are consequences of the traits microbes evolved to position themselves in a nutrient gradient, attach to a surface, and individually generate a flow of oxygen-rich water [16]. We identify three evolutionary innovations that allow cells to form veils. Cells must exert a minimum force, produce a mucous stalk and move to a particular concentration of a shared nutrient.

## 2. Material and methods

### 2.1 Collection of cells

Both *T. majus* and *Uronemella* cells were collected from the sulfidic mud of a salt marsh in Woods Hole, MA (40°31′33.34′′ N, 70°40′6.19′′ W). The techniques used to enrich these cells are nearly identical and have been previously described [16,29]. Inspection of water taken from pore spaces in this mud initially revealed an abundance of both *T. majus* and *Uronemella* living within the same samples. Over the course of three months of enrichment, *T. majus* cells became increasing rare and eventually vanished. *Uronemella* became increasingly abundant producing a clean enrichment culture. As the behaviours of *T. majus* individuals [17,18] and communities are very similar to those of *Uronemella* and *T. majus* is reported to grow as large as *Uronemella* [25], we incorrectly identified the ciliate as the larger morphology of *T. majus* in Petroff & Libchaber [16]. This was a mistake.

Enrichment cultures of *Uronemella*, containing no *T. majus*, have been grown and propagated for 2 years in salt water media [16,36] with a 10 mM agar plug, suggesting that this strain of *Uronemella* consumes free-swimming sulfur-oxidizing bacteria [18] or relies on sulfur-oxidizing epibionts or endobionts for energy.

### 2.2 Identification of *Thiovulum majus*

Because *T. majus* has so many unusual characteristics [25], it can be identified by its morphology and behaviour [5,12,13,27,37]. We identified *T. majus* by five aspects of its unusual morphology and behaviour. (i) *Thiovulum majus* is a very large bacterium, with a diameter of 5–25 μm [7,11,25]. The cells studied in this paper have a diameter of 8.5±0.5 μm [29]. (ii) *Thiovulum majus* contain sulfur globules concentrated in the posterior pole [12,25]. These globules are shown in figure 1*d*. (iii) As shown in figure 1*e*, these cells attach to surfaces with a stalk, organize into a dense band and form a veil. These behaviours are typical of *T. majus* [17]. (iv) As seen in the electronic supplementary material, video S1, unlike bacteria such as *Escherichia coli* (which divide longitudinally), these cells divide by elongating laterally, as has been previously reported for *T. majus* [11,30]. (v) The electron micrograph shown in figure 1*b* is quite similar to previously published micrographs [11], showing that the cell is covered with many short flagella. Additionally, we distinguished these cells from *Uronemella* with the DNA stain DAPI. Figure 1*c* shows that the chromosomes of these cells are distributed throughout the cell. By contrast, the chromosomes of *Uronemella* are distributed between the macronucleus and micronucleus (figure 2*c*).

### 2.3 Identification of *Uronemella*

DNA was extracted from the ciliate using a DNeasy Blood and Tissue Kit (Qiagen, Valencia, CA). 18S rRNA genes were amplified using the general eukaryotic primers MoonA (ACCTGGTTGATCCTGCCAG) and MoonB (TGATCCTTCYGCAGGTTCAC) [38]. PCR amplifications were done using PuReTaq Ready-To-Go PCR beads (GE Healthcare). The PCR thermal protocol (modified from [39]) was 3 min at 95°C followed by 35 cycles of: 30 s at 95°C (denaturation), 30 s at 57°C (annealing) and 2 min at 72°C (extension), with a final elongation step of 7 min at 72°C and a final holding of 4°C. The products were visualized on a 1% agarose gel. PCR products were purified with ExoSAP-IT (USB Corporation, Cleveland, OH). The PCR produced was sequenced directly at Laragen (Culver City, CA) using an AB3730XL with the corresponding PCR primers. Sequence trimming, editing and contig assembly were done manually in Sequencher (Gene Codes Coporation, MI). The 18S rRNA sequence was deposited in GenBank (accession number KT266872). BLAST [40] was used to determine the phylogenetic identity of the ciliate. Our sequence was 99% similar to two *Uronemella spp.* sequences in GenBank (accession numbers: HM236337.1, Gao *et al.* [41]; EF486866.1, Yi *et al.* [42]).

### 2.4 Electron microscopy

*Thiovulum majus* and *Uronemella* were fixed in 2.5% glutaraldehyde, washed with water and placed on aclar sheets coated with poly-l-lysine. They subsequently underwent the freeze-dry procedure reported in Santulli *et al*. [43] with minor modifications. Upon coating with iridium (ACE600, Leica), they were imaged on a Zeiss Leo 1550 using Zeiss InLens and SE2 secondary electron detectors.

### 2.5 Motion of *Uronemella* cells in an oxygen gradient

We observed the formation and dynamics of a *Uronemella* veil in an observation chamber constructed from a 330 μm-thick Thermo Scientific Gene Frame Seal (AB-0577) between a glass microscope slide and coverslip. Diffusion in the 330 μm gap between the glass slide and coverslip smooths oxygen gradients on the timescale of 50 s while the front of cells moves on the timescale of tens of minutes. Thus, oxygen gradients are effectively two-dimensional.

We observed these gradients with an oxygen sensitive fluorescent dye. The microscope slide was coated in the ruthenium-based dye tris(4,7-diphenyl-1,10-phenanthroline) ruthenium(II) dichloride (99% pure, American Elements RU-OM-02). The fluorescence of this dye is reversibly quenched by the presence of oxygen, allowing one to image the distribution of oxygen throughout the chamber [44,45]. The microscope slides were dip coated in a methyltriethoxysilane (Sigma Aldrich 175579) based sol-gel (3:1) doped with the oxygen-sensitive dye. Baking the dip coated slides for 144 h at 80° immobilizes the dye in a porous glass, through which oxygen diffuses [46,47]. To ensure that the dye is only deposited on the side of the slide with the chamber, we coated one side of the slide with nail polish. After dip coating, the nail polish was removed with acetone. We calibrated the oxygen detector with a two-point calibration to the Stern–Volmer relation [48].

At the beginning of the experiment, we inoculated free-swimming cells into the chamber. These cells were collected using a 1 ml pipette from a fresh *Uronemella* veil grown in the culture tubes described in Petroff & Libchaber [16]. We first lightly vortexed this material to break cells from the veil. The result was a mixture of free-swimming cells and broken pieces of veil. Removing the broken pieces of veil is vital to imaging the formation of a front as cells rapidly reattach to it, resulting in an inhomogeneous distribution of cells in the observation chamber. To remove the broken veil, the vortexed material was lightly centrifuged at 1500 r.p.m. (0.2*g*) for 90 s. This step separated the free-swimming cells from the dispersed veil material, which stuck to the centrifuge tube walls. We found this process to be more effective than filtration. We transferred 100 μl of media from the base of the centrifuge tube into the gene frame and sealed the top of the chamber with a cover slip. As the volume of the chamber was only 65 μl, extra inoculum spilled from the sides as the chamber was sealed, which prevented the inclusion of bubbles in the chamber. The final result was a uniform mixture of free-swimming cells in clear media at a concentration of approximately 5×10^4^ cells ml^−1^.

We imaged the distribution of cells and oxygen with a D5100 Nikon DSLR camera with a macro lens. The growth chamber was laid horizontally. A mirror below the chamber reflected an image of the chamber through a 570 nm-long pass glass filter (OG-570) to the camera. Two photos of the chamber were taken in rapid succession every 10 s. In the first photo, the sample was illuminated with a high-powered 455 nm LED (Thorlabs M455L3) to image the fluorescence of the oxygen-sensitive dye. The intensity of this photo was divided by the intensity of a photo of the chamber when saturated with oxygen. This normalization corrected for any small inhomogeneities in the distribution of dye. In the second photo, the sample was illuminated with a cold white high-powered LED (Thorlabs MCWHL5) to observe the scattered light from cells. To extract the cells from each of these images, we took the difference of sequential images to approximate the time derivative. As the cells move quickly, the time derivative of the image is large wherever the cells are moving.

## 3. Results and discussion

### 3.1 Overcoming diffusion

We begin by describing the advection and diffusion of nutrients around a cell and showing that a sessile cell must exert a minimum force to modify its chemical environment [49–51]. This scaling provides a rationale for the relatively large force produced by *T. majus*. Figure 3 shows the stream lines of water moving past a cell. The bacterium *T. majus* exerts a force on the surrounding fluid by rotating the hundreds of short flagella that cover the cell [11], as shown in figure 1*b*. When free swimming, the cell moves at a speed *U*_*t*_∼500 μm s^−1^ [27] and generates a force *f*_*t*_∼ 6*πμaU*_*t*_∼40 pN, where *μ* is the dynamic viscosity and *a* is the cell radius. By contrast, the ciliate *Uronemella* exerts a force with several hundred cilia, which, as shown in figure 2*b*, are arranged in lines extending from the cell anterior to posterior [15]. The cell beats these cilia coherently, generating waves of motion that pull water over the cell surface. Given a typical swimming speed *U*_*u*_∼250 μm s^−1^, we infer a force *f*_*u*_∼6*πμaU*_*u*_∼50 pN.

This force is exceptional by the standards of bacteria. Most bacteria, such as *E. coli*, exert a force *f*_0_∼1 pN [49,52,53]. Indeed, *T. majus* is one of the most powerful swimmers of the bacterial world [27]. We do not understand how these cells generate such a large force. In this section, we show that the viscosity of water and diffusivity of oxygen define a minimum force a cell must exert to overcome diffusion limitation. This constraint has led *T. majus* to become an unusually powerful swimmer.

When a stationary cell exerts a force on the surrounding fluid, the fluid at a distance *r* moves with velocity *u*∼*f*_0_/8*πμr* [20,50]. The resulting nutrient flux *j*_*a*_∼*uc*∼*f*_0_*c*/8*πμr*. As this flux carries nutrients to the cell, diffusion smooths nutrient gradients. The typical diffusive flux *j*_*d*_∼*Dc*/*r*. Taking the ratio of the advective to diffusive nutrient fluxes,

3.1 $$Pe=\frac{j_{a}}{j_{d}}=\frac{f_{0}}{8\pi D\mu }$$

is the Peclet number [49–51]. Thus, the influence of a filter-feeding cell on its chemical environment is determined by the ratio of the force it exerts to a diffusive force *f*_d_=8*πDμ*, which is determined by the viscosity of water and the diffusion coefficient of the relevant nutrient. Given a diffusion coefficient *D*∼10^−5^ cm^2^ s^−1^ typical of small molecules such as oxygen in water, we find *f*_d_∼25 pN. Both *T. majus* and *Uronemella* exert a force *f*_0_∼40 pN, yielding *Pe*≈2. If a cell applies a force significantly less than this value, diffusion smooths out nutrient gradients faster than they are maintained by advection.

Because *T. majus* generates a flow with flagella while *Uronemella* use cilia, we conclude that this similarity is the result of convergent evolution. Because the force needed is more than an order of magnitude larger than the force typical of bacteria, we conclude that natural selection has led *T. majus* to exert a power similar to that of *Uronemella*. By showing how the material properties of water lead filter-feeding microbes to exert similar forces, this scaling analysis provides our first example of convergent evolution.

### 3.2 Overcoming surface screening

The preceding scaling analysis places a limit on the force exerted by a filter-feeding cell, but it does not address how a filter-feeding cell attaches to a surface. When an attached cell pulls water towards it, the resulting flow is slowed as it moves over the surface. The surface screens the flow generated by an attached microbe, attenuating the nutrient flux to the cell.

*Thiovulum majus* and *Uronemella* respond to surface screening both individually and collectively. Individual cells overcome surface screening by exuding a stalk that allows the cell to position itself away from a surface. Communities of cells overcome surface screening by forming a permeable veil away from the marsh bottom. To understand how this collective behaviour arises from the individual behaviour of cells, here we consider the dynamics of an individual cell attached to an impermeable surface, such as a grain of sand.

The flow of water generated by a cell attached to a surface has been well studied both experimentally [21,51,54] and mathematically [55,56]. These papers consider the flow generated by a sessile cell [55] or one that moves with a prescribed motion [51,56]. Here, we couple the flow generated by a cell to the resulting cell motion to understand how a tethered cell orients itself. In this section, we provide a scaling analysis. In the following section, we derive the equations of motion.

**Figure 4.**
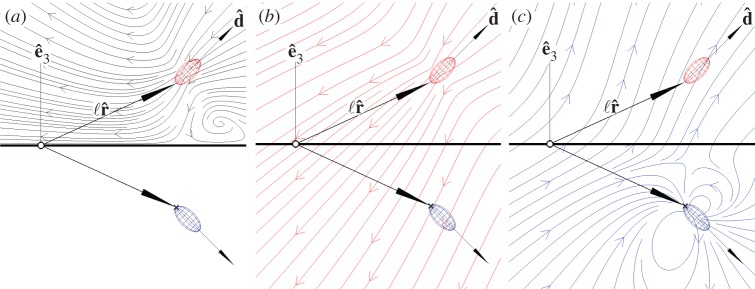
(*a*) The total flow of water (black stream lines) generated by the cell (red ellipsoid) near a boundary (thick black line) can be decomposed [20,50] into (*b*) the flow generated the cell in the absence of a boundary and (*c*) the back flow (blue arrows) from the surface due to an image cell (blue ellipsoid). This flow pushes the cell away from the surface.

We approximate a cell of size *a* as generating a point force of magnitude *f*_0_ oriented normal to a surface. As the cell pushes water towards the surface, there is a back flow that slows the flow. To leading order, the back flow from the surface can be approximated with an image cell (figure 4). Two additional higher order corrections are necessary to fully satisfy the no-slip boundary condition [20,50]. If the cell attaches directly to a surface, the resulting velocity field *u*_*a*_∼2*f*_0_*a*^2^/3*πμr*^3^, where *r*≫*a* is the distance from the cell [20]. However, if the cell separates itself from the surface by means of a stalk of length ℓ, the resulting velocity field *u*_ℓ_∼*f*_0_/8*πμr*, where *a*≪*r*≪ℓ. As this flow field decays much more slowly—decaying inversely with distance rather than the inverse cube—producing a stalk allows a cell to pull water towards it much more quickly than attaching to a surface. A more detailed analysis of the hydrodynamics of microbial filter-feeding is provided by Pepper *et al*. [21,51].

**Figure 5.**
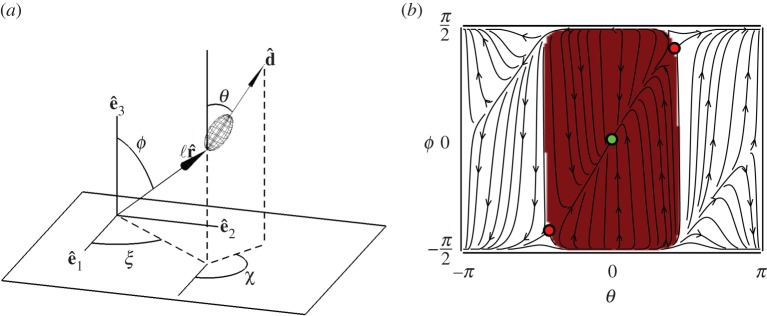
(*a*) Schematic of a cell anchored to a surface with a mucous stalk, of length ℓ and orientation $\hat{\text{r}}$. The cell has orientation $\hat{\text{d}}$. (*b*) Phase portrait showing the dynamics of a cell (with the aspect ratio of *Uronemella* and ℓ=10*a*) moving under the influence of the flow generated by its image cell. The dynamics admit a single stable fixed point (green point) and two unstable fixed points (red points). The basin of attraction (red shading) is roughly described by the strip −*π*/2<*θ*<*π*/2, implying cells initially oriented away from the surface will right themselves. If *ϕ*≈±*π*/2, the stalk lies flat on the surface. As the height of the cell above the surface approaches ℓ_*c*_, the cell becomes unstable; this leads to the curved corners of the basin of attraction. As *T. majus* is more spherical than *Uronemella*, ℓ_*c*_ is correspondingly smaller, leading to a basin of attraction with slightly sharper corners but otherwise identical.

It is not obvious that simply producing a stalk allows a cell to move away from a boundary rather than crashing into it. To understand the motion of a cell anchored to a surface, we consider the fluid forces that cause the cell to align with its stalk and the stalk perpendicular to the surface [51]. First, when the cell is not oriented parallel to its stalk, the torque on the cell pulls its stalk to align its body and stalk. As the cell exerts a force $f_{0}\hat{\text{d}}$, it pulls its stalk with a torque ${\text{L}}_{\text{s}}=f_{0}\ell \hat{\text{r}}\times \hat{\text{d}}$. Consequently, the cell moves through the fluid with velocity **u**, yielding a viscous torque ${\text{L}}_{\text{v}}=-\ell \hat{\text{r}}\times 6\pi \mu a\text{u}$. Balancing these torques and taking *u*∼ℓ/*τ*_*f*_, we find that the cell orients itself with its stalk on a timescale *τ*_*f*_∼6*πμa*ℓ/*f*_0_≈0.5 s. Next, as the cell pushes water downwards towards the surface, there is a back flow off the surface that pushes the cell upward (figure 5c). The back flow *u*_*s*_∼*f*_0_/8*πμ*ℓ pushes the cell body away from the surface on a timescale *τ*_*s*_∼ℓ/*u*_*s*_≈6 s. Because *τ*_*f*_/*τ*_*s*_∼*a*/ℓ≈0.1 is small, the cell quickly aligns with its stalk and then, more slowly, moves away from the surface.

This scaling analysis shows how developing a stalk allows a cell to separate itself from a surface and thereby generate a much larger flow. These dynamics provide a physical basis for the convergent evolution of these microbes. The stalks produced by *T. majus* and *Uronemella* attach to the cell in substantially different manners. *Thiovulum majus* exudes mucus from a specialized feature on the cell wall [11]. By contrast, the *Uronemella* stalk derives from the caudal cilium [15]. These anatomical differences imply that the qualitative similarities of stalk formation are the result of convergent evolution rather than, for example, horizontal gene transfer.

### 3.3 Equations of motion for a tethered cell

In this section, we derive the equations of motion for a tethered cell anchored to a surface by a stalk. We find that these dynamics admit a single stable fixed point in which the cell is oriented normal to the surface provided the length of the stalk is longer than a critical value, which is of order one cell length. Unlike the rest of this paper, this section assumes the reader has a working familiarity with low Reynolds number hydrodynamics. As the ecologically relevant physical phenomena revealed by these dynamics are captured in the preceding scaling analysis, this section may be treated as an appendix.

This derivation is presented in two steps. First, we consider how a cell, which is anchored to a surface by a stalk, moves in response to an arbitrary ambient flow. Next, we consider the special case that the ambient flow is generated by the image cell (i.e. the back flow off the surface).

Because the cell cannot accelerate, the torque vanishes at the point where the stalk meets the surface to which the cell is attached. The total torque at this point results from two forces acting on the cell. The cell exerts a force $-f\hat{\text{d}}$ that, when $\hat{\text{d}}$ is not aligned with $\hat{\text{r}}$, causes the cell to move through the water. This force is balanced by hydrodynamic drag as the cell moves relative to the background fluid velocity **u**. The resulting torque balance requires

3.2 $$\text{0}=\hat{\text{r}}\times \left\{f\hat{\text{d}}+6\pi \mu a\text{A}\left(\text{u}-\ell \frac{\partial \hat{\text{r}}}{\partial t}\right)\right\}.$$

Because the cell is not spherical, the drag coefficient depends on orientation of the cell relative to the flow. The matrix $\text{A}=\alpha_{∥}\hat{\text{d}}\hat{\text{d}}+\alpha_{⊥}(\text{I}-\hat{\text{d}}\hat{\text{d}})$ decomposes the flow into the part parallel to $\hat{\text{d}}$, with drag coefficient *α*_∥_, and the part orthogonal to $\hat{\text{d}}$, with drag coefficient *α*_⊥_ [50]. **I** is the identity matrix. Given the aspect ratio *a*/*b*∼1.3, typical of *T. majus*, *α*_∥_=0.82 and *α*_⊥_=0.86. For the slightly more elongated *Uronemella* cell, *a*/*b*∼2, yielding *α*_∥_=0.60 and *α*_⊥_=0.69.

Next, we require that the cell be torque free. There are three elements of this torque balance. First, because the stalk does not attach at the cell centre, a cell moves through the water as it rotates about the stalk. The torque **L**_1_ required to translate a prolate ellipsoid along a circular arc of radius *a* relative to a flow **u** is

3.3 $${\text{L}}_{1}=\hat{\text{d}}\times \left\{6\pi \mu a\text{A}\left(\text{u}-a\frac{\partial \hat{\text{d}}}{\partial t}\right)\right\}.$$

The second element of the torque balance represents the drag on the cell as it rotates with an angular velocity ***ω*** different from the ambient angular velocity ***Ω***. The resulting torque [50,57,58] is

3.4 $${\text{L}}_{2}=8\pi \mu a^{3}\text{B}(\Omega -\omega ),$$

where the matrix $\text{B}=\beta_{∥}\hat{\text{d}}\hat{\text{d}}+\beta_{⊥}(\text{I}-\hat{\text{d}}\hat{\text{d}})$ relates the drag on the cell to the rotation of the cell relative to $\hat{\text{d}}$ [50]. A prolate ellipsoid with the aspect ratio of a *T. majus* cell has drag coefficients *β*_⊥_=0.65 and *β*_∥_=0.54 for rotation perpendicular and parallel to $\hat{\text{d}}$, respectively. In the case of *Uronemella*, *β*_⊥_=0.38 and *β*_∥_=0.20. Finally, if the viscous stress imposed by the flow passing over the cell is not distributed symmetrically over the cell, the stress twists the cell. Given a stress field ***τ***, the resulting torque [50,57,58] on the cell is

3.5 $${\text{L}}_{3}=8\pi a^{3}\beta_{⊥}\Gamma (\hat{\text{d}}\times \tau \cdot \hat{\text{d}}),$$

where *Γ*=(*a*^2^−*b*^2^)/(*a*^2^+*b*^2^)≈0.26 (*T. majus*) or *Γ*≈0.60 (for *Uronemella*) describes how prolate (*Γ*>0) or oblate (*Γ*<0) the cell is. The total torque balance on the cell requires that

3.6 $$\text{0}={\text{L}}_{1}+{\text{L}}_{2}+{\text{L}}_{3}.$$

Following Pedley & Kessler [58], because $∥\hat{\text{d}}∥=1$ the angular velocity can be expressed as $\omega =\omega_{∥}\hat{\text{d}}+\hat{\text{d}}\times \partial \hat{\text{d}}/\partial t$. Taking the vector product of equation (3.6) with $\hat{\text{d}}$ and simplifying gives the equation of motion for the cell orientation:

3.7 $$\frac{\partial \hat{\text{d}}}{\partial t}=-\frac{\hat{\text{d}}\times \{3\hat{\text{d}}\times \text{A}\text{u}+4a\hat{\text{d}}\times \text{B}\Omega +4a\beta_{⊥}\Gamma (\hat{\text{d}}\times \text{E}\cdot \hat{\text{d}})\}}{a(3\alpha_{⊥}+4\beta_{⊥})},$$

where **E**=***τ***/*μ* is the rate of strain field. This equation relates the changes in the cell orientation $\hat{\text{d}}$ to the local flow (**u**, ***Ω*** and **E**), drag coefficients (**A**, **B**, *α*_⊥_ and *β*_⊥_), cell shape *Γ* and cell size *a*. Notably, if *a* is small compared to all relevant scales, the influence of the angular velocity ***Ω*** and stress are negligible.

These equations of motion (3.2) and (3.7) admit a single stable fixed point. At this fixed point, the cell and stalk are oriented normal to the surface. It follows from the symmetry of the system that a cell so oriented will not move. The stability of this fixed point can be understood intuitively from figure 5*c*. Provided the cell is oriented away from the surface (i.e. ${\hat{\text{e}}}_{3}\cdot \hat{\text{d}}>0$), the flow due to the image bacterium tends to push the cell away from the boundary, thus reorienting the cell and stalk towards the surface normal ${\hat{\text{e}}}_{3}$.

One slight modification to this motion becomes apparent by writing the equation of motion for the polar angle *θ* of the cell. The definition of this angle is shown in figure 4. Simplifying equation (3.7) and taking the flow field of the image cell as the ambient flow, we find

3.8 $$t_{s}\frac{\partial \theta }{\partial t}=\frac{9}{16}\frac{\beta_{⊥}a\Gamma (3+\cos2\theta )-3\ell \alpha_{⊥}\cos\theta \cos\phi }{a(3\alpha_{⊥}+4\beta_{⊥})\cos^{2}\phi }\sin\theta ,$$

where *t*_*f*_=6*πμa*ℓ/*f*∼0.5 s. From this equation, we see that the fixed point *θ*=0 is stabilized by the term proportional to tether length ℓ and destabilized by the term proportional to cell size *a*. The stabilizing term reflects the tendency of the cell to align with the ambient flow (equation (3.3)), which was the intuitive foundation of the previous stability argument. The destabilizing term arises from the stress over the cell surface (equation (3.5)), which twists the cell towards the image cell. Because the stabilizing term is proportional to the tether length, for ℓ≫*a* the effect of drag dominates and the cell remains oriented normal to the surface.

To find the minimum length of a tether required for a cell to orient normal to a surface, we calculate the eigenvalues of the linearized equations of motion near the fixed point. These equations of motion have a stable fixed point at which a cell orients normal to the surface provided that

3.9 $$\ell >\ell_{c}=\frac{4\beta_{⊥}\Gamma }{3\alpha_{⊥}}a.$$

We find ℓ_*c*_/*a*=4*β*_⊥_*Γ*/(3*α*_⊥_)≈0.26 and ℓ_*c*_/*a*≈0.44 for *T. majus* and *Uronemella*, respectively. As stalks are always substantially longer then the cell, both types of cell orient away from the surface. The dynamics of cells with stalks shorter than ℓ_*c*_ are additionally modified by the finite size of the cell and interactions between the boundary and the flagella/cilia.

Finally, we investigate the nonlinear motion of a cell near a boundary by numerically integrating the equations of motion. Because the system is rotationally symmetric, the dynamics of the azimuthal angles *χ* and *ξ* (defined in figure 5*a*) are trivial. Thus, the dynamics of the cell are determined entirely by the evolution of the two polar angles *ϕ* and *θ*. The phase portrait of these dynamics are shown in figure 5*b*. Within the basin of attraction (red shading in figure 5*b*) of the single stable fixed point, the dynamics can be usefully decomposed into two parts. The largest torque on the system is due to the misalignment of the cell with the stalk. Thus, the fastest motion in the phase portrait is towards the line *ϕ*=*θ*. Once the cell and stalk are aligned, the flow due to the image bacterium pushes the cell away from the surface.

### 3.4 Collective dynamics of cells generate a large-scale fluid flow

In this final section, we consider the collective dynamics of *T. majus* and *Uronemella*. We begin by reviewing what is known about the tendency of *T. majus* to accumulate at a particular oxygen concentration [17,18] and provide new, complementary measurements of *Uronemella* accumulation. We then measure the rates of stalk elongation and the rate at which stalks accumulate into veil. Finally, for completeness, we briefly review the stability of a veil [16] and discuss the similar collective dynamics previously observed in *T. majus* and *Uronemella* veils.

**Figure 6.**
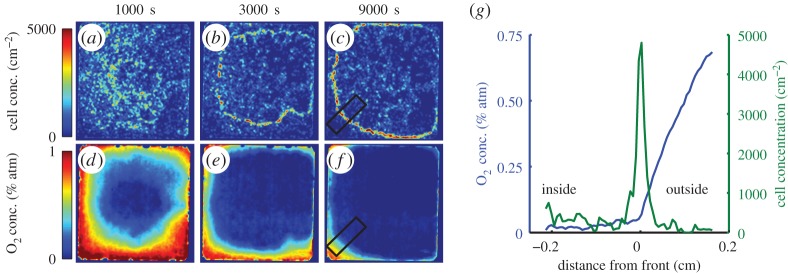
The *Uronemella* veil forms at a constant oxygen concentration. Panels (*a*–*c*) show four snapshots of the density of cells in the observation chamber. Over the course of the experiment, the cells reorganize from a uniform distribution into a dense front with a roughly circular form. Each panel is 1.5 cm on a side. Panels (*d*–*f*) show the distribution of oxygen in the chamber. The front forms at a constant oxygen concentration and separates the oxygen-poor interior of the chamber from the oxygen-rich borders. The chamber edges are oxygen permeable. Panel (*g*) shows the average density of cells (green) and oxygen concentration (blue) around the front as measured within the black rectangles in panels (*c*,*f*). Most of the cells are concentrated within a dense front. The change in the slope of the oxygen profile is due to cell metabolism.

During the first step of veil formation, cells respond by accumulating into a dense front at a preferred oxygen concentration. This behaviour is well known in both *T. majus* [17] and in several microaerophilic ciliates [59–61] including species of *Uronemella* [18]. Fenchel has shown that *T. majus* cells are chemotactic to a particular oxygen concentration *c*_*t*_=4% of the atmospheric value. As cells accumulate at this value, they form a dense band. Figure 6 shows that the ciliate *Uronemella* behaves in the same way. Panels (*a*–*c*) show snap shots of cell density at different times after the inoculation of cells into the chamber. Over the course of an hour, cells concentrate into a dense front of cells. This front grows slowly outwards from the centre of the chamber to the boundaries. Panels (*d*–*f*) show the distribution of oxygen through the chamber as the front forms and moves. At the beginning of the experiment, the media is well mixed. Oxygen diffuses into the chamber through the gas-permeable chamber walls and is consumed by the cells. After 1000 s, cells consume oxygen producing an oxygen minimum in the centre of the chamber. After 3000 s, the moving front separates the low oxygen interior from the high oxygen exterior. As shown in panel (*g*), the front forms at a particular concentration *c*_*u*_=7% atmospheric concentration. Thus, we conclude that the organization of *Uronemella* cells, like *T. majus*, relies on chemotaxis to self-organize into a front. Videos of the formation of the front and evolution of the oxygen field are provided as the electronic supplementary material.

*Thiovulum majus* and *Uronemella* differ from many other microaerophilic cells in their abilities to generate mucous stalks and produce a powerful flow. As shown in the previous sections, these traits allow them to individually mitigate diffusion limitation and viscous dissipation. We now show how these traits allow cells to produce a veil and cooperate to generate a macroscopic flow.

**Figure 7.**
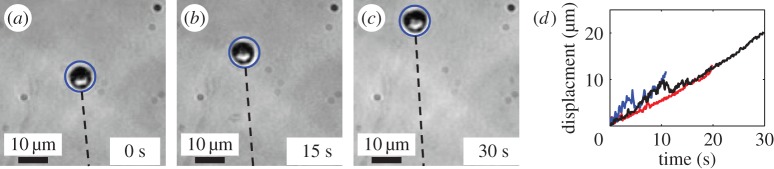
The stalk of a *T. majus* slowly elongates. Panels (*a*–*c*) show the position of a cell (blue circle) and stalk (dashed line) at 15 s intervals. The stalk position is estimated from the cell orientation. (*d*) The displacement of three isolated cells all increase as the stalk elongates at a typical speed 0.65 μm s^−1^.

**Figure 8.**
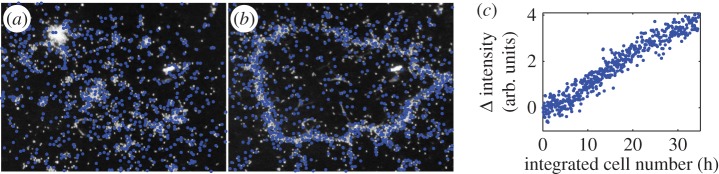
*Uronemella* weave stalks into a veil. *Uronemella* organize into a front and produce veil at a constant rate. Panels (*a*–*b*) show two images of the distribution of cells (blue dots) and veil (white material) within the chamber. The cells form a closed veil. Each panel is 1 cm across. (*c*) The intensity of light scattered from the veil increases as cells produce new material. The change in scattered light intensity (*Δ* intensity) is proportional to the integrated cell number. Thus, each cell produces veil material at a constant rate, consistent with figure 7.

Cells continue to produce new stalk material when anchored to a surface. When either a *T. majus* or an *Uronemella* cell anchors to a surface, it continues to produce extracellular polymeric substances. Consequently, the stalk slowly elongates. To measure the rate of stalk elongation, we observed the position of cells anchored to a glass slide as a function of time for between 10 and 30 s. As shown in figure 7, *T. majus* stalks grow at a speed of approximately 0.65 μm s^−1^. *Uronemella* stalk grows at a similar speed of approximately 1.5 μm s^−1^. This material builds up in the environment. Where the cells are dense, this material accumulates and provides cells new surfaces to which they attach. This phenomenon is particularly apparent in *Uronemella*, as these cells form relatively thick stalks that readily scatter light. It is not necessary to stain the veil to observe it. Figure 8*a*,*b* shows two images of an *Uronemella* community as cells come together to form a veil. Cells are marked with blue dots. The white material shows veil produced from the entwined broken stalks of cells. Cells preferentially attach to the edges of the existing veil. Given that the stalk of each cell elongates at a constant rate, the total rate of veil production is proportional to the number of cells. Thus, the total amount of veil material is proportional to the number of cells integrated over time. We define this quantity as the integrated cell number. As shown in figure 8*c*, the intensity of scattered light is indeed proportional to the integrated cell number. Thus, veil is produced at a constant rate as the cell stalks accumulate in the surrounding water.

Because cells within a front are all competing with one another, they quickly become limited by the availability of oxygen. If the front is stationary, the oxygen flux decreases in time *t* as $j∼Dc/\sqrt{Dt}$ as diffusion causes oxygen gradients to smooth out. To remain at the optimal oxygen concentration, cells must generate a flow of water that pulls oxygen through the community faster than the diffusive flux.

Counterintuitively, simply forming a veil is not sufficient to generate a macroscopic flow. If cells are uniformly distributed over a veil, the flow created by each cell exactly cancels the flow of each other cell [23,24]. This balance follows directly from the incompressibility of water. As cells on a veil push water towards an impermeable surface, the total flow towards the surface must balance the flow away. By symmetry, a uniform distribution of cells therefore produces no flow. Thus, uniformly distributed cells only stir the water on the scale of a single microbe. Cells benefit from collective behaviour by organizing non-uniformly on a veil. By concentrating into patches, cells attached to a veil generate a convective flow on a scale determined by the distance between patches [9,22–24]. Because diffusion is much less efficient than advection in transporting material over large distances, the onset of density fluctuations corresponds to a dramatic increase in the Peclet number.

**Figure 9.**
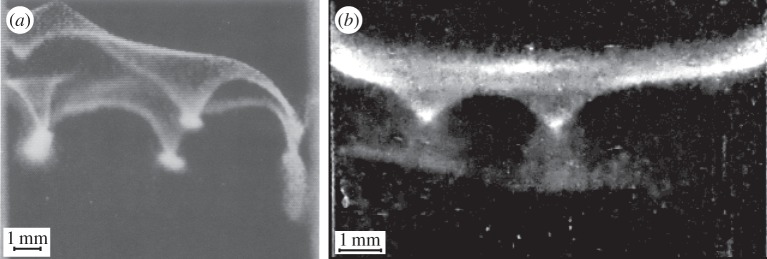
Veils produced by (*a*) *T. majus* and (*b*) *Uronemella* generate similar density fluctuations, called dimples. Panel (*a*) is adapted with permission from [27]. *Uronemella* dimples shown in (*b*) are described in Petroff & Libchaber [16].

Figure 9 shows that veils produced by *T. majus* and *Uronemella* develop very similar large-scale density fluctuations called ‘dimples’. The dimples shown in figure 9a were observed in a *T. majus* veil that formed in a 3-ml glass cuvette of width approximately 1 cm [27]. The *Uronemella* dimples shown in figure 9*b* formed in a glass test tube of diameter 1.1 cm [16].

Dimples form as cells redistribute themselves over the veil surface. The onset of density fluctuations and flow generation have been previously described [16]. We briefly review this instability to highlight how these macroscopic dynamics arise due to the formation of mucous stalks and large force exerted by each cell. Because cells attach to the veil with a mucous thread, stalks periodically break, causing cells to swim away from the veil. These cells turn around, returning to the veil to at a random point typically within a few hundred micrometres from the point of detachment [17,18]. This process of attachment and detachment from the veil causes individual cells to perform a random walk over the veil surface. As cells are chemotactic to a preferred oxygen concentration, they diffuse more slowly where oxygen is closer to the optimal [17,18]. Thus, the dynamics of dimple formation have some similarity to the run-and-tumble random walks of bacterial chemotaxis [62,63]. However, there are two important differences. First, cells only move over the two-dimensional veil surface rather than through the three-dimensional fluid. Second, because these cells are powerful swimmers, living at *Pe*∼2, cells modify oxygen gradients as they move in response to them. The steady-state density of cells in a dimple generates a flow large enough to balance the metabolic rate of cells at the optimal oxygen concentration. The steady-state shape of the dimple is determined by the forces acting on the veil as cells push water through it. In addition to these viscous forces, the veil is also shaped by gravitational (veil is denser than water) and elastic forces. The final shape of the dimple reflects the balance of gravitational and viscous forces, which tend to deform the veil, with elastic forces.

## 4. Conclusion

The bacterium *T. majus* and the ciliate *Uronemella* have independently evolved the ability to form a veil. This is a remarkable behaviour. Hundreds of thousands or even millions of cells come together to create an elastic membrane that may extend for thousands of body lengths. The cells then organize relative to one another on this membrane to stir the surrounding fluid. This behaviour does not require an equally complex ensemble of adaptations to allow each cell to control and coordinate its motion. This behaviour relies on just three traits, each of which benefits the individual. First, cells must be powerful swimmers. The viscosity of water and the diffusion coefficient of oxygen define a typical force an individual cell must exert to draw oxygen from its environment faster than the diffusive flux. This limit has forced *T. majus* to become one of the most powerful swimmers of the bacterial world [27]. Second, microbes living in the diffusive boundary layer must attach to a surface. Individual *T. majus* and *Uronemella* produce a stalk that positions the cell away from the surface [11,12,15–18], thus limiting surface screening. Because these cells continuously produce new material to replace broken stalks, this material accumulates in the environment as a veil. Third, these microaerophilic cells move to a particular oxygen concentration. Consequently, cells form a dense front where broken stalks accumulate into a veil. As cells attach and detach from the veil, they organize to generate a macroscopic flow.

Although *T. majus* and *Uronemella* form veils through the same dynamics, we have never observed a veil composed of a mixture of these microbes. We suggest two explanations. First, *T. majus* and *Uronemella* accumulate at slightly different concentrations of oxygen (table 1). Consequently, if these microbes were grown together in a gradient, *Uronemella* would form a veil at a slightly higher concentration than *T. majus*. As the *Uronemella* veil consumes the oxygen, it may inhibit the formation of the *T. majus* veil. Alternatively, *Uronemella* may tend to consume *T. majus*, thus preventing veil formation. Both of these explanations suggest the appearance of *Uronemella* and of *T. majus* veils in nature require spacial inhomogeneities that separate populations of *T. majus* and *Uronemella*.

It is useful to consider how the ubiquitous limitations of diffusive transport and surface screening are reflected in the traits of two microbes that do not form veils. First, *Stylonychia* produces a flow while attached directly to a surface [64]. However, rather than exerting a force normal to the surface (like *T. majus* and *Uronemella*), this microbe exerts a force parallel to the surface [64]. This cell orientation minimizes the effect of surface screening for a cell attached directly to a surface. *Vorticella* generates a *Pe*∼1 flow and produces a proteinaceous stalk that anchors the cell to a surface [21,51,64,65]. Because these stalks are proteinaceous rather than mucous, cells rarely replace broken stalks. Consequently, this material does not accumulate to form a veil.

The focus of this work has been to understand how the similar collective behaviour of veil-forming microbes is a reflection of the similar traits these microbes have evolved to overcome diffusion limitation and mitigate surface screening. The evolutionary history that led to these traits remains unclear. The flagella of *T. majus* are composed of the same material [30] as those of *E. coli* yet it is able to exert a force an order of magnitude greater. Future work should seek to understand how this bacterium is able to generate such a large flow. The production of the mucous stalk is also mysterious. Future work should seek to understand how *T. majus* and *Uronemella* exude material to form a single thread rather than an encasing matrix.

## Supplementary Material

 figure_data.tar.gv provides all of the data plotted in the manuscript and a read_me file

## Data accessibility

The 18S rRNA sequence of *Uronemella* was deposited in Genbank (accession number KT266872). The data for each figure are provided in the electronic supplementary material.
